# H2BE113K mutation promotes breast cancer metastasis through modulating chromatin dynamics

**DOI:** 10.1126/sciadv.adx4982

**Published:** 2026-07-10

**Authors:** Shiman Hu (胡诗曼), Jiaxian Liu, Jiaqi Zhou, Jiaohua Chen, Tiantian Qin, Yi Ching Esther Wan, Xiaoxuan Zhu, Danyi Wang, Chuting Shao, Yabin Chen, Xin Wang, Junhong Han, Hoi Leong Xavier Wong, Robert S. Weiss, Haojie Jin, Mo Chen, Qing Li, Yogen Saunthararajah, Haiyun Gan, Kui Ming Chan (陈居明)

**Affiliations:** ^1^ Department of Biomedical Sciences, College of Biomedicine, City University of Hong Kong, Hong Kong SAR, China.; ^2^ Key Laboratory of Biochip Technology, Biotech and Health Centre, Shenzhen Research Institute of City University of Hong Kong, Shenzhen, China.; ^3^ Institute of Biochemistry and Molecular Biology, Guangdong Provincial Key Laboratory of Medical Immunology and Molecular Diagnostics, the First Dongguan Affiliated Hospital, Guangdong Medical University, Dongguan, China.; ^4^ Shenzhen Key Laboratory of Synthetic Genomics, Guangdong Provincial Key Laboratory of Synthetic Genomics, Key Laboratory of Quantitative Synthetic Biology, Shenzhen Institute of Synthetic Biology, Shenzhen Institutes of Advanced Technology, Chinese Academy of Sciences, Shenzhen, China.; ^5^ Department of Surgery, Li Ka Shing Institute of Health Sciences, The Chinese University of Hong Kong, Hong Kong SAR, China.; ^6^ Department of Biotherapy, Cancer Center and State Key Laboratory of Biotherapy, Frontiers Science Center for Disease-related Molecular Network, West China Hospital, Sichuan University, Chengdu, China.; ^7^ School of Chinese Medicine, Hong Kong Baptist University, Hong Kong SAR, China.; ^8^ Department of Biomedical Sciences, Cornell University, Ithaca, NY, USA.; ^9^ State Key Laboratory of Oncogenes and Related Genes, Shanghai Cancer Institute, Renji Hospital, Shanghai Jiao Tong University School of Medicine, Shanghai, China.; ^10^ State Key Laboratory of Molecular Oncology, School of Basic Sciences, Tsinghua University, Beijing, China.; ^11^ State Key Laboratory of Protein and Plant Gene Research, School of Life Sciences and Peking-Tsinghua Center for Life Sciences, Peking University, Beijing, China.; ^12^ Department of Translational Hematology and Oncology Research, Taussig Cancer Institute, Cleveland Clinic, Cleveland, OH, USA.

## Abstract

Cancer progression is driven by the accumulation of DNA mutations and aberrant gene regulation. Recent studies have demonstrated that multiple H3 mutations serve as drivers of tumorigenesis. However, the role and significance of various cancer-associated histone H2B mutations in cancer development remain unknown. Here, we investigate H2BE113K, a missense mutation of histone H2B predominantly found in patients with breast cancer. We show that H2BE113K promotes colony formation in breast cancer. Notably, transcriptomic analysis reveals differential expression of genes in various cancer pathways in H2BE113K cells. The loci with elevated gene expression display increased chromatin accessibility, accompanied by H2BE113K enrichment. Depletion of *G3BP2*, one of the H2BE113K target genes that has been implicated in breast cancer, reduces the colony formation phenotype in H2BE113K cells. In addition, H2BE113K knock-in mice crossbred with an MMTV-PyMT breast cancer model show elevated lung metastasis. Together, our findings provide critical insights in the mechanistic role of H2BE113K in gene regulation, chromatin function, and breast cancer progression.

## Teaser

H2BE113K regulates cancer-related gene expression by modulating chromatin dynamics and promotes breast cancer metastasis.

## INTRODUCTION

Histones are nuclear proteins essential for DNA packaging and epigenetic gene regulation (*1*). Two copies of each core histone (H2A, H2B, H3, and H4) wrapped around with 147 bp of DNA to form a nucleosome (*2*). Over the past decade, mutations in genes encoding histones have been identified in various diseases, especially in cancers (*3*). Given the critical roles of these cancer-associated histone mutations in cancer progression, their protein products are referred to as “oncohistones.” Notably, recent research has revealed that histone mutations are present in ~3.8% of all tumor samples, which is comparable to the incidence of mutations in nonhistone cancer-associated genes within the same cohort (*4*, *5*). These findings indicated that the significance of histone mutations was underestimated and highlighted the importance of deciphering the mechanisms of oncohistones in cancer development.

Mutations occurring in histone H3, including H3K27M, H3G34V/R, and H3K36M, are the most well-studied cancer-related histone mutations and have been shown to play significant roles in cancer progression. Of note, H3K27M and H3G34V/R were first characterized in 2012 (*6*, *7*) and have been elucidated to play critical roles in diffuse intrinsic pontine gliomas (DIPGs) (*8*, *9*). In addition, H3K36M mutation was reported to play a vital role in head and neck squamous cell carcinomas and chondroblastoma (*10*–*12*). The H3K27M and H3K36M mutations share similar mechanisms, contributing to cancer development through the misregulation of the corresponding lysine posttranslational modifications. We and others have demonstrated that H3K27M mutation disrupts the posttranslational modification of lysine at position 27 of histone H3, subsequently reprograms the epigenetic landscape, alters gene expression, and eventually leads to tumorigenesis (*8*, *9*, *13*).

In addition to H3, a significant number of H2B histone mutations were identified in patients with cancer (*14*). We previously investigated the roles of H2BG53D and H2BE76K mutations in pancreatic cancer and breast cancer, respectively (*15*–*18*). Another histone H2B mutation, H2BE113K, was identified predominantly in breast and lung cancers. Of note, this mutation occurs at the Glu^113^ (E113) residue of histone H2B, which resides within the nucleosome acidic patch (*19*, *20*). The nucleosome acidic patch consists of eight residues: E56, E61, E64, D90, E91, E92 of H2A, and E105 and E113 of H2B (*21*) and interacts with histone binding proteins containing “arginine anchors” (*22*, *23*). The chromatin remodeling complex families, including SWItch/Sucrose Non-Fermentable (SWI/SNF) (*24*, *25*) and Imitation SWItch (ISWI) (*26*–*28*), are the primary proteins that were shown binding to the nucleosome acidic patch and are essential for the nucleosome dynamics, including nucleosome assembly and sliding (*29*, *30*). Despite the shared domains and structures among chromatin remodeler core subunits, distinct functional roles have been identified for each chromatin remodeler (*31*, *32*). Among these complexes, ISWI is one of the four major chromatin remodelers in mammalian cells (*33*). Notably, ISWI regulates transcription and numerous biological processes through mediating nucleosome sliding and altering chromatin accessibility (*34*–*36*). Although in vitro studies have shown that H2BE113K increased the activity of SMARCA5, the functional unit with the adenosine triphosphatase activity of the ISWI chromatin remodeler complex (*37*, *38*), the biological relevance and molecular mechanism of H2BE113K in cancer development remain poorly understood.

Breast cancer is one of the most frequently diagnosed cancers and remains a leading cause of cancer-related mortality among women globally. Despite advancements in early detection and treatment, significant challenges persist, including variability in disease progression and drug resistance (*39*). The mouse mammary tumor virus–polyoma middle T antigen (MMTV-PyMT) model is one of the most widely used animal models for breast cancer research. This transgenic mouse model exhibits short tumor latency and lung metastasis (*40*), making it a valuable genetic model for investigating the molecular mechanisms of breast cancer progression. In this study, we used CRISPR-Cas9 and gene targeting approaches to generating the H2BE113K knock-in breast cancer cells and H2BE113K knock-in mice. With these genetically modified models, we investigated the mechanistic roles of the H2BE113K mutation in breast cancer in vitro and in vivo. Our findings demonstrate the critical roles of H2BE113K in chromatin regulation, cellular function, and breast cancer progression.

## RESULTS

### H2BE113K promotes colony formation in breast cancer cells

**Fig. 1.**
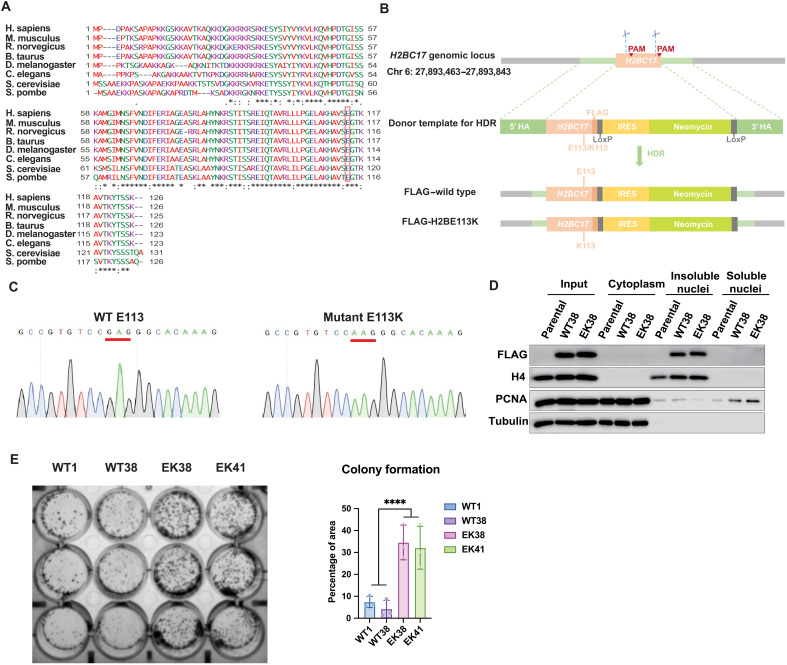
H2BE113K promotes colony formation in breast cancer cells. (**A**) H2BE113 is evolutionary conserved from yeast to higher organisms. Multiple sequence alignment of H2B proteins from different species. Glutamic acid (E) at position 113 of human H2B protein is marked by a red rectangle. (**B**) Schematic diagram showing the gene targeting strategy of generating FLAG-H2BWT and FLAG-H2BE113K knock-in MDA-MB-231 cell lines. PAM, protospacer adjacent motif; IRES, internal ribosome entry site; HDR, homology-directed repair; 5' HA, 5' homology arm. (**C**) Sequencing chromatograms confirmed the E to K mutation at the position 113 of H2B. (**D**) H2BE113K mutation of H2B did not affect the recruitment of H2B to the chromatin. Cell extracts were separated into cytoplasm, insoluble nuclei, and soluble nuclei fractions. Immunoblotting was performed with indicated antibodies. Representative images from three independent experiments are shown. PCNA, proliferating cell nuclear antigen. (**E**) Representative images of colony formation assay showing that H2BE113K mutant cells acquired higher colony formation ability. Quantification analysis from three independent experiments are shown. *P* value was calculated by unpaired, two-tailed Student’s *t* test (mean ± SD, ****P < 0.0001).

The glutamic acid (E) at position 113 of histone H2B is one of the eight residues forming the acidic patch of the nucleosome (*20*, *41*). Recent reports from Bagert *et al.* (*37*) and Dao *et al.* (*38*) have revealed the effects of cancer-associated mutations of these eight residues on nucleosome spacing and chromatin remodeling in vitro. However, how H2BE113K, the most frequent mutation among the residues of the nucleosome acidic patch, affects cellular function remains largely unknown. In addition, the role of H2BE113K in cancer development in vivo is still unclear. As expected, the H2BE113 residue is evolutionarily conserved from yeast to higher eukaryotes including mammals (Fig. 1A), indicating that the E113 residue is crucial for histone H2B and nucleosome functions. Analysis of The Cancer Genome Atlas (TCGA) database via cBioPortal identified two types of H2BE113 missense mutations, the “H2BE113-to-K” and “H2BE113-to-Q.” The H2BE113K mutation was found in a variety of tumor types, including six cases of breast invasive ductal carcinoma, as well as in lung, head and neck, and bladder cancers. In contrast, H2BE113Q mutation was identified in a single case of breast cancer (fig. S1, A and B). Therefore, we knocked-in the H2BE113K mutation into the invasive human breast cancer cell line MDA-MB-231, to investigate the effect of this mutation on gene regulation and cancer phenotype.

We used a CRISPR-Cas9 approach to knocking-in the E113K mutation into a single allele of one of the 14 H2B genes in MDA-MB-231 cells to express the E113K mutant H2B protein at physiological levels mimicking patient conditions (Fig. 1B). The *H2BC17* gene was selected since single guide RNAs (sgRNAs) targeting this H2B gene had the highest predicted specificity scores, and mutation at E113 in *H2BC17* was observed in a patient with cancer. Upon CRISPR-Cas9–mediated genome editing and homology-directed repair, DNA sequences encoding FLAG-tagged wild-type (WT) or E113K mutated histones were integrated into the *H2BC17* locus. We took this opportunity to add a FLAG tag at the C terminus for detecting the expression and mapping the WT and E113K mutant H2B to the genome. The gene targeting outcomes of independent WT and E113K mutant clones were confirmed by Sanger sequencing and polymerase chain reaction (PCR) genotyping (Fig. 1C). Hereinafter, the knock-in cell lines expressing the FLAG-WT H2B and FLAG-E113K H2B are referred to as WT and H2BE113K cells, respectively.

To determine the effect of H2BE113K on chromatin integration, we firstly examine the endogenous FLAG expression level of WT and H2BE113K mutant cells. Our results showed that the expression level of the FLAG-tagged E113K-H2B was comparable to the FLAG-tagged WT-H2B in isogenic clones (Fig. 1D). Moreover, by cell fractionation assay, we demonstrated that the H2BE113K mutation did not affect the deposition of histone H2B to the chromatin (Fig. 1D). Although the H2BE113K mutation had no effect on cell proliferation and cell migration (fig. S1, C and D), it significantly enhanced colony formation ability by breast cancer cells (Fig. 1E).

### H2BE113K alters chromatin accessibility and gene regulation

**Fig. 2.**
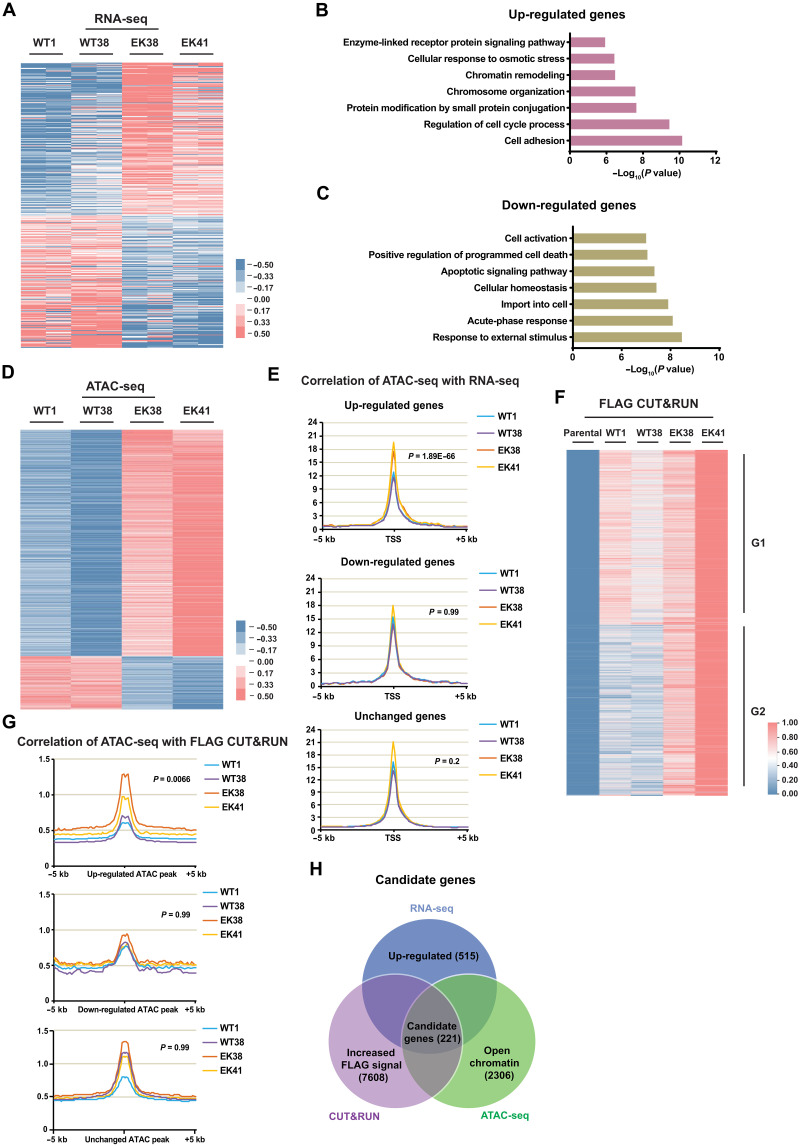
H2BE113K alters chromatin accessibility and gene regulation. (**A**) Heatmap showing the DEGs (defined with *P* < 0.01) in H2BE113K mutant cells relative to WT cells. (**B** and **C**) Histograms revealing the top gene ontology (GO) terms enriched among genes that are significantly up-regulated (B) and down-regulated (C) in the H2BE113K mutant cells. *P* values were calculated by a hypergeometric test. (**D**) Heatmap demonstrating differential chromatin accessibility between WT and mutant cells. (**E**) Density plots revealing normalized read density of ATAC-seq data for gene categorized on the basis of expression alterations from RNA-seq. (**F**) Heatmap showing differential FLAG CUT&RUN signals between WT and H2BE113K mutant cells. The unchanged and increased FLAG peaks in mutant cells versus WT were divided into G1 and G2 groups, respectively. (**G**) Density plots illustrating normalized read density of FLAG CUT&RUN for peaks categorized on the basis of ATAC-seq data. (**H**) Venn diagram showing the overlapped genes that are up-regulated in RNA-seq, enriched with FLAG signal in FLAG CUT&RUN, and presented high chromatin accessibility in ATAC-seq in H2BE113K cells.

To investigate how H2BE113K enhanced colony formation in breast cancer, we first performed RNA sequencing (RNA-seq), and our results identified hundreds of differentially expressed genes (DEGs) in the H2BE113K mutant cells compared to the WT isogenic cells (Fig. 2A). Gene ontology and pathway analysis showed that the up-regulated genes in H2BE113K cells were enriched in multiple cancer-related pathways, while the genes involved in extracellular matrix organization pathways were down-regulated (Fig. 2, B and C). Since the nucleosome acidic patch is implicated in chromatin dynamics, we performed assay for transposase-accessible chromatin sequencing (ATAC-seq) to examine whether the differential expression of genes in H2BE113K mutant cells was correlated with alterations in chromatin accessibility (Fig. 2D). Motif analysis of differential ATAC-seq peaks identified distinct transcription factor binding motifs, indicating that the H2BE113K mutation creates a more permissive chromatin landscape for specific transcriptional regulators (fig. S2A). Analysis of ATAC-seq and RNA-seq data integration revealed that increased chromatin accessibility was primarily associated with up-regulated gene expression (Fig. 2E). Specifically, 45.8% of up-regulated genes showed increased chromatin accessibility. Notably, 69% of regions with increased accessibility were linked to genes that did not show significant expression changes. Furthermore, altered chromatin accessibility was a rare event among down-regulated and unchanged genes, occurring in ~4% of these genes (Fig. 2E).

To further elucidate the role of H2BE113K in chromatin regulation, we performed FLAG cleavage under targets and release using nuclease sequencing (CUT&RUN-seq) to map the genomic localization of both the FLAG-tagged WT H2B and E113K mutant H2B. MDA-MB-231 parental cells were used as negative control for FLAG CUT&RUN, while H3 CUT&RUN was performed to serve as internal control. Analysis of peak distribution revealed that 50.8% of FLAG-E113K peaks colocalized with FLAG-WT H2B (referred to the G1 cluster), while 49.2% were specific to the E113K mutant (G2 cluster; Fig. 2F). Integrating our ATAC-seq data with the FLAG CUT&RUN results revealed that 9440 ATAC-seq peaks overlap with FLAG peaks (14.7% of total ATAC-seq peaks; fig. S2B). Moreover, the peaks with increased chromatin accessibility exhibited higher FLAG enrichment (G2 cluster; Fig. 2G and fig. S2C). Notably, 93% of these increased accessibility peaks overlapped with the G2 cluster. Furthermore, our results demonstrated that genes within the G2 cluster exhibited a twofold greater incidence of increased chromatin accessibility (10%) than those in the colocalized G1 cluster (5%) (fig. S2D). These results suggested that the H2BE113K mutation might play a crucial role in altering gene expression via chromatin regulation.

To investigate the molecular mechanism by which the H2BE113K enhanced colony formation in breast cancer cells, we further analyzed the genes that showed (i) specific enrichment of FLAG-E113K H2B (cluster G2 in Fig. 2F), (ii) elevated chromatin accessibility, and (iii) up-regulation in H2BE113K mutant cells. The genes down-regulated in H2BE113K cells were not further analyzed as they exhibited neither changes in chromatin accessibility nor specific alterations in FLAG enrichment. We identified 269 loci including 221 genes plus their alternative splicing variants (Fig. 2H) and characterized their gene functions (fig. S2, E and F). Multiple cancer-related pathways were identified, and some of the candidate genes have been implicated in various cancers, including *RAD50* and *G3BP2* (fig. S2F).

### Up-regulation of *G3BP2* in H2BE113K cells enhances colony formation

**Fig. 3.**
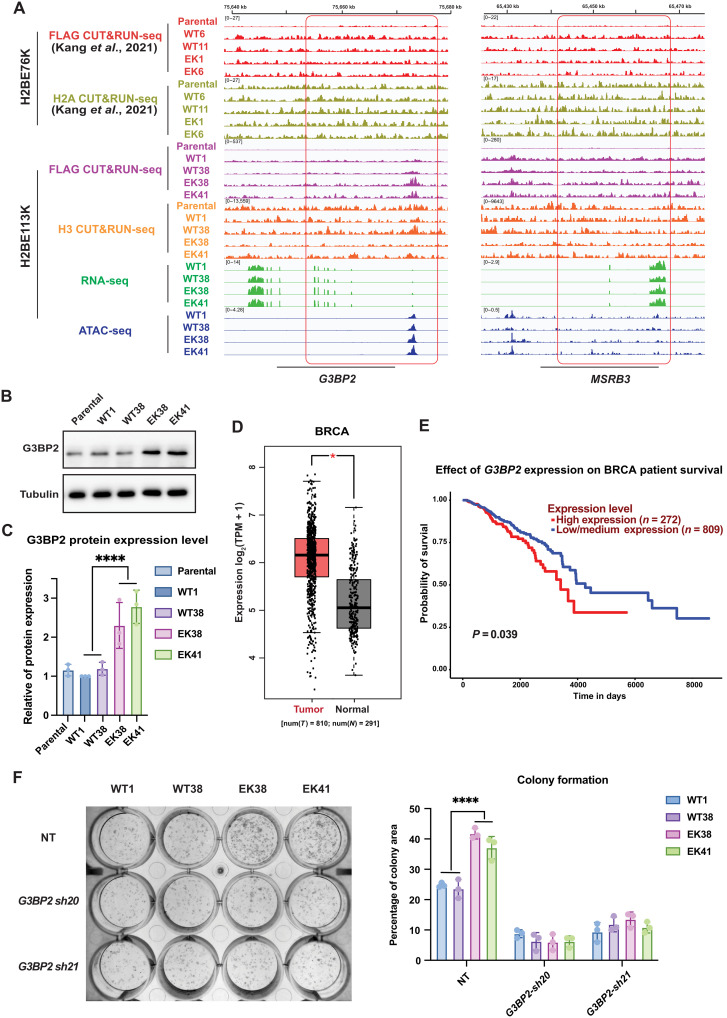
Up-regulation of *G3BP2* in H2BE113K cells enhanced colony formation. (**A**) Genome browser tracks of FLAG, H3, and H2A CUT&RUN-seq, RNA-seq, and ATAC-seq data at the *G3BP2* (left) and its nearby gene loci (right). Upper tracks revealing the FLAG & H2A CUT&RUN-seq results obtained from WT and H2BE76K mutant cells previously published by us [Kang *et al.* (*17*)]. Lower tracks illustrating the FLAG/H3 CUT&RUN-seq, RNA-seq, and ATAC-seq results from WT and H2BE113K mutant cells. (**B**) Immunoblotting demonstrating G3BP2 protein levels in parental, WT, and H2BE113K knock-in cells. Representative images from three independent experiments are shown. (**C**) Quantification analysis showing protein level of G3BP2 in parental, WT, and mutant cells. Results from three independent experiments are shown. *P* value was calculated by unpaired, two-tailed Student’s *t* test (mean ± SD, ****P < 0.0001). (**D**) Expression levels of *G3BP2* in tumors and normal tissues in patients with breast invasive carcinoma (BRCA). Data were extracted from the TCGA database and plotted through the Gene Expression Profiling Interactive Analysis (GEPIA) online tool. TPM, transcripts per million. (**E**) Kaplan-Meier (KM) plot revealing *G3BP2* expression–based overall survival analysis. The KM plot was generated by web-based tool UALCAN on patients with breast cancer. (**F**) Colony formation assay showing the impact of *G3BP2* knockdown in WT and H2BE113K mutant cells. Results from three independent experiments are shown. *P* value was calculated by unpaired, two-tailed Student’s *t* test (mean ± SD, ****P < 0.0001).

Among the 221 candidate genes, G3BP stress granule assembly factor 2 (*G3BP2*) caught our attention since it has been implicated in breast cancer in recent studies (*42*, *43*). To this end, we chose *G3BP2* as an example to decipher how H2BE113K affects breast cancer progression via altering gene regulation and chromatin function. The FLAG CUT&RUN and ATAC-seq data indicated the enrichment of E113K-H2B and increased chromatin accessibility at *G3BP2* locus (Fig. 3A). The specificity of FLAG enrichment at *G3BP2* in H2BE113K mutant cells was further validated by (i) comparison with our previous FLAG CUT&RUN from MDA-MB-231 cells with H2BE76K knock-in mutation (Fig. 3A) (*17*) and (ii) evaluation of the *MSRB3* locus which locates nearby *G3BP2* and displayed comparable FLAG signals in WT and H2BE113K mutant cells (Fig. 3A). The elevated expression of *G3BP2* in H2BE113K mutant cells was shown by RNA-seq (Fig. 3A) and confirmed by Western blotting and quantitative reverse transcription PCR (RT-qPCR; Fig. 3, B and C, and fig. S3A). Our results revealed that *G3BP2* expression was significantly up-regulated in H2BE113K mutant cells at both mRNA (1.5-fold, *P* = 0.0029) and protein levels (2.3-fold, *P* < 0.0001). Higher expression of *G3BP2* was also found in breast cancer samples (Fig. 3D) and was linked to poorer prognosis (Fig. 3E), demonstrating the clinical relevance of the expression level of *G3BP2* in breast cancer. To further investigate the role of the *G3BP2* in the enhanced colony formation induced by H2BE113K, *G3BP2* was depleted in both WT and E113K mutant cells (fig. S3, B and C). Of note, our results demonstrated that short hairpin RNA (shRNA) knockdown of *G3BP2* significantly reduced the colony formation ability of both WT and the E113K mutant cells. Abrogation of *G3BP2* in E113K mutant cells resulted in cell viability comparable to that of the non-targeting (NT) group of WT cells (Fig. 3F), further revealing the significance of the *G3BP2* level in the oncogenic effects of H2BE113K mutation.

### H2BE113K regulates *G3BP2* by recruiting SMARCA5

**Fig. 4.**
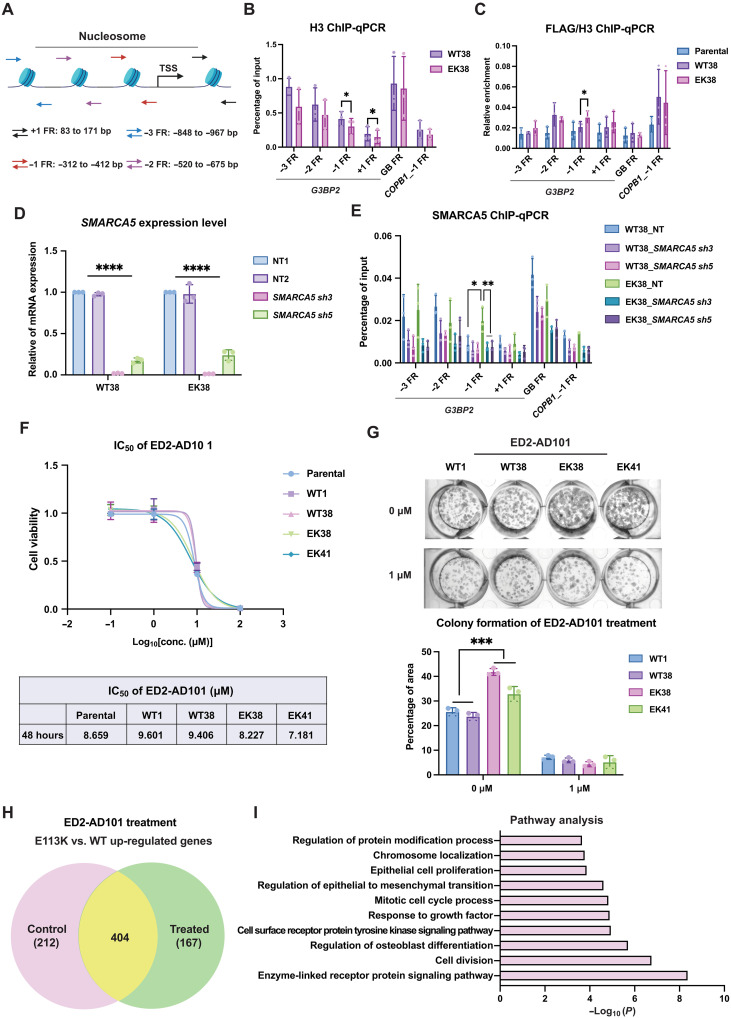
H2BE113K regulates *G3BP2* by recruiting SMARCA5. (**A**) Schematic showing the locations of the primers used in ChIP-qPCR relative to the nucleosome position and TSS of *G3BP2*. FR, forward and reverse. (**B**) ChIP-qPCR analysis of H3 enrichment at various genomic regions, including −3, −2, −1, and + 1 nucleosomes and gene body (GB) of *G3BP2* and −1 nucleosome of *COPB1*. Results from three independent experiments are shown (mean ± SD, *P < 0.05). (**C**) ChIP-qPCR analysis of relative FLAG enrichment at indicated gene loci. FLAG enrichment over H3 was calculated as fold change relative to H3 ChIP. Results from three independent experiments are shown (mean ± SD, *P < 0.05). (**D**) RT-qPCR analysis revealing mRNA levels of *SMARCA5* in WT and H2BE113K mutant cells after shRNA-mediated knockdown. Results from three independent experiments are shown (mean ± SD, ****P < 0.0001). (**E**) ChIP-qPCR analysis of SMARCA5 enrichment at various genomic regions, including −3, −2, −1, and + 1 nucleosome and GB of *G3BP2* and −1 nucleosome of *COPB1.* Results from three independent experiments are shown (mean ± SD, *P < 0.05 and **P < 0.01). (**F**) Cytotoxicity of ED2-AD101 was evaluated using the CCK8 assay in the knock-in cell lines and parental cells. IC_50_, median inhibitory concentration. (**G**) Colony formation assay showing the impact of ED2-AD101 in WT and H2BE113K mutant cells. Representative images from three independent experiments are shown. Bottom: quantification of colonies formed after ED2-AD101 treatment in cells. Results from three independent experiments are shown (mean ± SD, ***P < 0.001). (**H**) Venn diagram demonstrating the numbers of up-regulated genes in H2BE113K mutant cells in the control or ED2-AD101 treated group. (**I**) Histograms revealing the top GO terms and Kyoto Encyclopedia of Genes and Genomes pathways enriched among the 212 genes specifically up-regulated in the H2BE113K mutant cells compared to WT cells from the control group.

To validate the increased chromatin accessibility at the *G3BP2* loci in H2BE113K cells as shown by our ATAC-seq results (Fig. 3A), we performed chromatin immunoprecipitation (ChIP) assays using histone H3 antibody. Our ChIP-qPCR results revealed a reduction of histone H3 occupancy at the transcription start site (TSS) and the promoter region (denoted by the −3, −2, and −1 nucleosomes) (Fig. 4, A and B, and fig. S4A) but not in the gene body of *G3BP2* or other genes (*COPB1*) that were not targeted by H2BE113K (fig. S3D). FLAG ChIP-qPCR results showed significant enrichment of FLAG-tagged E113K mutant H2B at the −1 nucleosome position in E113K mutant cells when normalized to H3 (Fig. 4C and fig. S4B). These findings suggested a preferential enrichment of E113K mutant H2B and a reduction of histone occupancy at the promoter and TSS regions of *G3BP2*.

Recent studies have shown that the E113K mutant H2B enhanced the enzymatic activity of SMARCA5 in vitro (*37*, *38*). To test whether SMARCA5 plays a role in modulating *G3BP2* expression in E113K mutant cells, we first examined the expression level of *SMARCA5* in WT and E113K mutant cells. Our results showed that H2BE113K mutant cells had similar expression of *SMARCA5* at both protein and mRNA levels compared to WT isogenic cells (fig. S4, C and D). Our ChIP-qPCR results with *SMARCA5* depleted cells as controls revealed a significant enrichment of SMARCA5 at the −1 nucleosome position of *G3BP2*, but not at other tested regions in E113K mutant cells (Fig. 4, D and E, and fig. S4, E and F). These results indicated that SMARCA5 was enriched at the *G3BP2* promoter region, where it might play a role in regulating chromatin accessibility and altering *G3BP2* expression in H2BE113K mutant cells.

Since SMARCA5 is enriched at the *G3BP2* promoter in H2BE113K mutant cells, we tested whether inhibiting SMARCA5 might influence cellular function of mutant cells. Notably, the SMARCA5 inhibitor ED2-AD101 (*44*) had no effect on the expression levels of *SMARCA5* and *G3BP2* (fig. S4G). Although the half-maximal inhibitory concentration (IC_50_) of ED2-AD101 was similar in WT and H2BE113K mutant cells (Fig. 4F), ED2-AD101 exhibited a profound inhibitory effect on colony formation by H2BE113K mutant cells compared with WT cells (Fig. 4G). To elucidate the molecular mechanism underlying differential response to ED2-AD101, RNA-seq was performed in both WT and H2BE113K mutant cells. Our transcriptomic analysis identified various DEGs that were specifically up-regulated or down-regulated in H2BE113K cells upon ED2-AD101 treatment compared to WT cells (Fig. 4H and fig. S4H). Pathway analysis revealed that ED2-AD101 treatment eliminated the up-regulation of cell division–related genes, which was observed in the H2BE113K control group (Fig. 4I). Moreover, ED2-AD101 treatment resulted in the down-regulation of multiple genes in the H2BE113K mutant cells compared to WT cells, many of which were implicated in the cellular homeostasis (fig. S4I). These findings demonstrated that ED2-AD101 suppressed the enhanced colony formation induced by H2BE113K mutation. Moreover, although the effect of ED2-AD101 might not be dependent on *G3BP2*, these results highlighted the therapeutic potential of SMARCA5 inhibition for patients harboring H2BE113K mutation.

### H2BE113K promotes lung metastasis of breast cancer in vivo

**Fig. 5.**
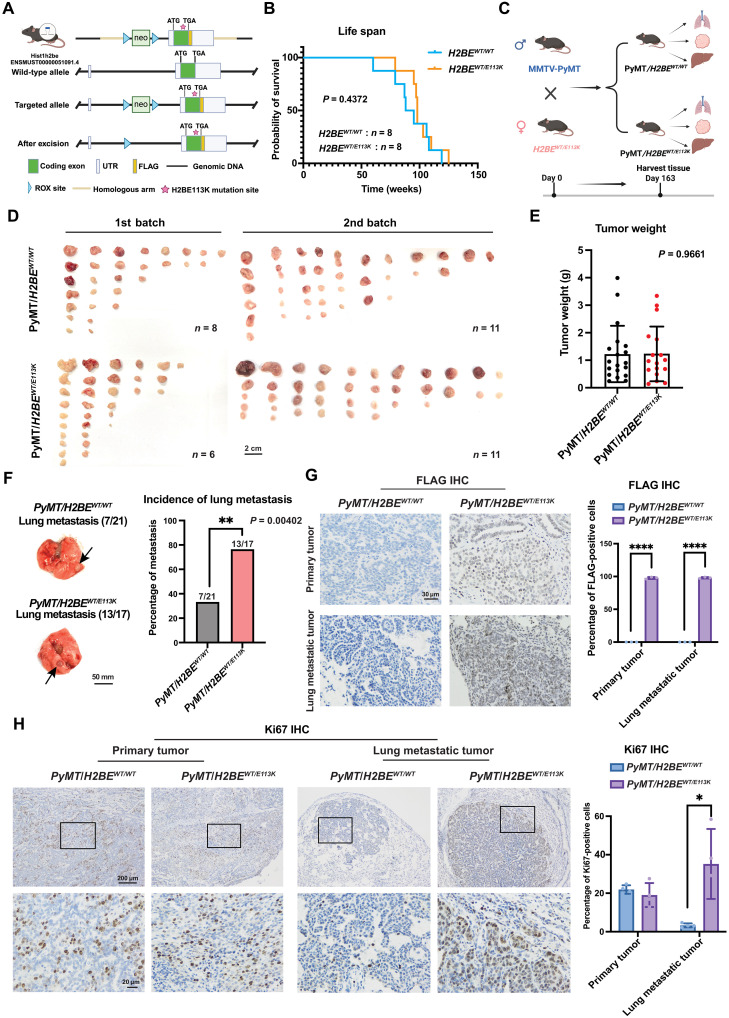
H2BE113K promoted lung metastasis of breast cancer in vivo. (**A**) Schematic of the gene targeting strategy of generating H2BE113K knock-in mouse. (**B**) KM survival curves of *H2BE^WT/WT^* and *H2BE^WT/E113K^* mice. *P* value was calculated using a log-rank test. (**C**) Schematic of breeding strategy and workflow. (**D**) Images and (**E**) quantification of the weight of primary tumors harvested from two batches of *PyMT/H2BE^WT/WT^* (*n* = 19) and *PyMT/H2BE^WT/E113K^* (*n* = 17) mice. Scale bar, 2 cm. *P* value was calculated by an unpaired, two-tailed Student’s *t* test. (**F**) Representative images (left) and quantification (right) of lung metastases harvested from *PyMT/H2BE^WT/WT^* and *PyMT/H2BE^WT/E113K^* mice. *P* value was calculated by a *z*-score test. Scale bar, 50 mm. The arrows indicated the lung metastatic tumors. (**G**) Representative images (left) and quantification (right) of FLAG IHC staining of primary tumors and lung metastatic tumors harvested from *PyMT/H2BE^WT/WT^* and *PyMT/H2BE^WT/E113K^* mice. The FLAG-positive cells were counted, and a *P* value was calculated by unpaired, two-tailed Student’s *t* test (mean ± SD, ****P < 0.0001). (**H**) Representative images (left) and quantification (right) of Ki67 IHC staining of primary tumors and lung metastatic tumors harvested from *PyMT/H2BE^WT/WT^* and *PyMT/H2BE^WT/E113K^* mice. The Ki67-positive cells were counted, and a *P* value was calculated by unpaired, two-tailed Student’s *t* test. (mean ± SD, *P < 0.05).

Our cell culture data revealed that the H2BE113K mutation enhanced colony formation of breast cancer cells but did not address the impact of H2BE113K on breast cancer progression. To evaluate the role of H2BE113K in cancer development in vivo, we generated H2BE113K knock-in mice in the C57BL/6 background. The *H2BE* gene, one of the 19 actively expressed H2B genes in the mouse genome, was selected for gene targeting. A FLAG tag was inserted at the C terminus of the E113K mutant H2B protein for tracking its expression (Fig. 5A). As expected, our results demonstrated that heterozygous H2BE113K mutation, “*H2BE^WT/E113K^*” did not affect the growth, development, and the life span of mice in both sexes (Fig. 5B and fig. S5, A and B). To examine whether the H2BE113K mutation affects breast cancer development, we crossbred the H2BE113K mice with the mammary-specific polyomavirus middle T antigen overexpression (MMTV-PyMT) breast cancer mouse model (Fig. 5C). We observed no significant difference in tumor latency or overall survival between *PyMT/H2BE^WT/WT^* and *PyMT/H2BE^WT/E113K^* female mice (fig. S5C). Although the onset and the development of primary mammary tumors were not affected by the H2BE113K mutation (Fig. 5, D and E, and fig. S5, D and E), we found a significant increase in the incidence of lung metastasis in the *PyMT/H2BE^WT/E113K^* mice (Fig. 5F).

Hematoxylin and eosin (H&E) staining of primary and lung metastatic tumors revealed no morphological difference between *PyMT/H2BE^WT/WT^* and *PyMT/H2BE^WT/E113K^* mice (fig. S5, F and G). To evaluate the H2BE113K signal distribution, immunohistochemistry (IHC) using the FLAG antibody was applied. The staining results revealed that the FLAG signal presented specifically in the *PyMT/H2BE^WT/E113K^* but not *PyMT/H2BE^WT/WT^* mammary tissues and tumor samples, indicating that the H2BE113K was expressed in the *PyMT/H2BE^WT/E113K^* cells (Fig. 5G). Although Ki67 expression was comparable between the primary tumors of *PyMT/H2BE^WT/WT^* and *PyMT/H2BE^WT/E113K^* mice, we observed a significantly higher Ki67 level in the lung metastatic tumors of *PyMT/H2BE^WT/E113K^* mice compared to *PyMT/H2BE^WT/WT^* mice (Fig. 5H). Collectively, these findings demonstrated that the H2BE113K mutation promoted lung metastasis of breast cancer, likely a consequence of higher cell proliferation.

### H2BE113K regulates metastasis-related genes and pathways of breast cancer in vivo

**Fig. 6.**
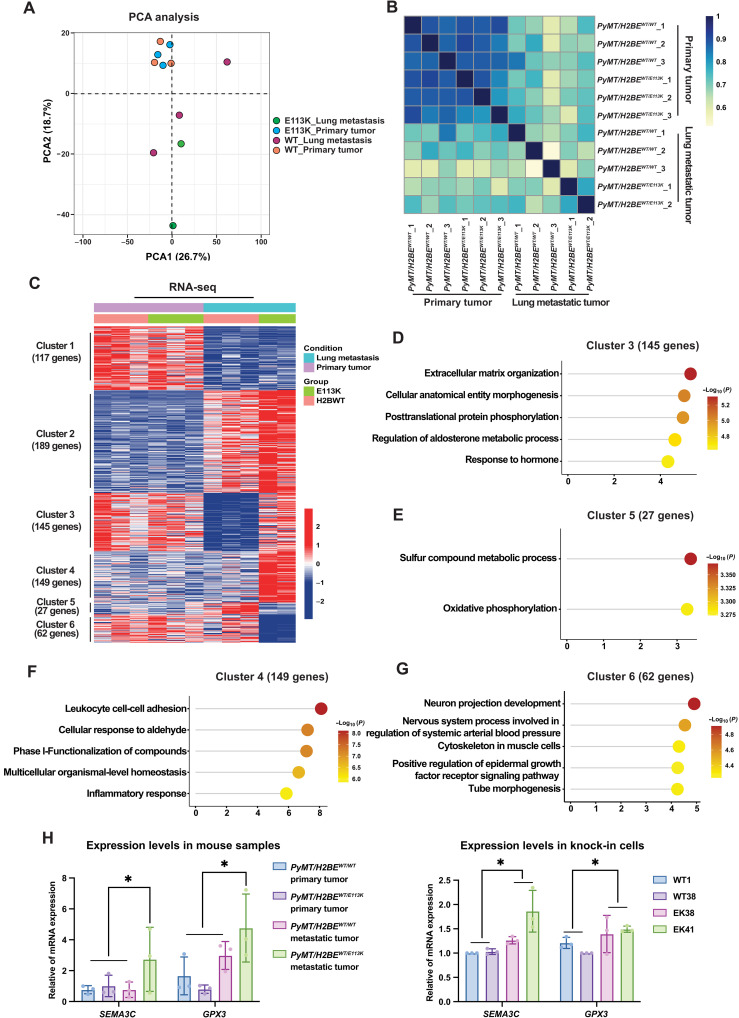
H2BE113K regulates metastasis-related genes and pathways of breast cancer in vivo. (**A**) PCA analysis of RNA-seq results of the tumor samples, including primary tumors and lung metastatic tumors harvested from *PyMT/H2BE^WT/WT^* and *PyMT/H2BE^WT/E113K^* mice. Biological replicates are highlighted with the same color. (**B**) Similarity matrix analysis shown as a heatmap, with colors representing the Pearson correlation between different samples listed in (A). (**C**) Heatmap showing the DEGs between various groups of tissue samples. DEGs were categorized into six clusters, with gene number indicating in each cluster. (**D** to **G**) The bubble plots illustrating the top enriched GO terms within the molecular function category for genes in clusters 3 to 6. The color gradient represented the −log_10_ (*P* value). The threshold for significance was set as a *P* value < 0.05, and the GO terms were ranked on the basis of their enrichment scores. (**H**) RT-qPCR analysis revealing mRNA levels of *SEMA3C* and *GPX3* in tumor samples (left) and knock-in cell lines (right). Results from three independent experiments are shown (mean ± SD, *P < 0.05). *P* value was calculated by an unpaired, two-tailed Student’s *t* test.

Our histological analysis of lung metastases revealed the elevated expression of cell proliferation marker Ki67 in *PyMT/H2BE^WT/E113K^* mice; however, the molecular mechanism underlying the increased lung metastasis remained elusive. To decipher the underlying molecular basis, we performed RNA-seq on primary tumors and lung metastases collected from *PyMT/H2BE^WT/WT^* and *PyMT/H2BE^WT/E113K^* mice. Principal components analysis (PCA) and similarity matrix analysis revealed comparable transcriptomic profiles between the primary tumors of the *PyMT/H2BE^WT/WT^* and *PyMT/H2BE^WT/E113K^* mice (Fig. 6, A and B). In contrast, a significant difference was observed in the lung metastases of *PyMT/H2BE^WT/E113K^* compared to *PyMT/H2BE^WT/WT^* mice. Pathway analysis of the RNA-seq data revealed differential expression of genes in various cancer pathways in the lung metastases of the *PyMT/H2BE^WT/E113K^* mice (Fig. 6, C to G).

We categorized the DEGs into six clusters according to their expression patterns (Fig. 6C). In particular, cluster 1 and cluster 2 included metastasis-related genes that were consistently up-regulated or down-regulated in lung metastatic samples compared to primary tumors. Notably, cluster 3 and cluster 5 represented the genes specifically down-regulated (145 genes) or up-regulated (27 genes) in the *PyMT/H2BE^WT/WT^* lung metastases while exhibiting opposite expression patterns in all other samples. Moreover, pathway analysis highlighted several metastatic related pathways, which associated with the genes in cluster 3 and cluster 5, including extracellular matrix organization (Fig. 6, D and E) and cellular entity morphogenesis. Our results indicated that these genes might play a role in promoting H2BE113K-driven metastasis.

Cluster 4 and cluster 6 featured genes were uniquely up-regulated (149 genes) or down-regulated (62 genes) in *PyMT/H2BE^WT/E113K^* lung metastases, indicating that these genes might contribute to the enhancement of the lung metastasis process induced by H2BE113K. These DEGs were enriched in pathways including leukocyte cell-cell adhesion, suggesting their critical role in accelerating the lung metastasis driven by H2BE113K mutation (Fig. 6, F and G). We further correlated the gene expression profiles of lung metastatic tumors (fig. S6A) in *PyMT/H2BE^WT/E113K^* mice (Fig. 6C) to the H2BE113K knock-in MDA-MB-231 cells (Fig. 2A). We found multiple overlapping genes, including *SEMA3C* and *GPX3*, indicating their critical roles in the phenotype induced by H2BE113K in both in vitro and in vivo. Previous studies have shown that semaphoring 3C (*SEMA3C*) played a significant role in the breast cancer development. *SEMA3C* was highly expressed in breast cancer, and its elevated expression was correlated with poor prognosis (fig. S6, B and C), and the knockdown of *SEMA3C* significantly impeded the proliferation and migration ability of breast cancer cells (*45*, *46*). Last, we examined the expression levels of these overlapping genes in mouse tumor samples and knock-in cell lines of WT and H2BE113K genetic background by RT-qPCR. Our data confirmed that both the H2BE113K mutant cells and lung metastatic tumors of *PyMT/H2BE^WT/E113K^* mice showed higher expression levels of *SEMA3C* and *GPX3* compared to WT cells and other tumor samples (Fig. 6H and fig. S6D). In summary, these results demonstrated that H2BE113K mutation accelerates lung metastasis of breast cancer by regulating the expression of metastatic-related gene.

## DISCUSSION

Recent studies by us and others have revealed the roles and mechanisms of various histone H3 mutations in cancer development. However, the functional relevance of histone H2B mutations remained largely unclear. In this study, we elucidated the mechanisms of H2BE113K, a missense mutation of histone H2B substituting lysine for glutamic acid at position 113, in breast cancer development. We used two distinct models to study H2BE113K: (i) FLAG-tagged WT/H2BE113K knock-in MDA-MB-231 human breast cancer cells and (ii) FLAG-tagged H2BE113K knock-in mice in the C57BL/6 background. Although the two models are largely different in terms of genetic composition (human cell line versus mouse) and breast cancer subtypes (triple-negative basal-like versus luminal B) (*47*), we identified multiple overlapping genes that showed the same differential expression patterns in these two models. One of the target genes, *SEMA3C*, has been shown to play a critical role in breast cancer development. We deciphered a direct role of H2BE113K in regulating the expression of target genes in our cell model by tracing the genomic localization through FLAG CUT&RUN. However, the same analysis is not feasible in our animal model since a mouse strain with FLAG-tagged WT H2B gene is not available.

The enhanced colony formation by H2BE113K mutant cells in vitro partially reflected the increased lung metastasis phenotype observed in *PyMT/H2BE^WT/E113K^* mice. *PyMT/H2BE^WT/E113K^* mice did not show enhanced primary tumor growth compared to *PyMT/H2BE^WT/WT^* controls, supporting the idea that the H2BE113K mutation, which has a low incidence in breast cancer, may play a role at the late stage during breast cancer progression. As reported in cohorts from Memorial Sloan Kettering cancer center (MSK) (*48*) and TCGA (*49*–*53*) datasets, the H2BE113K mutation was found in ~0.6% of all breast cancer cases. Although the H2BE113K mutation occurs at a low incidence, considering the substantial global burden of breast cancer (note that 2.3 million women were diagnosed with breast cancer, with 670,000 deaths reported in 2022), there are still a significant number of patients harboring this mutation, highlighting the importance of investigating the underlying mechanisms of the H2BE113K mutation.

Unexpectedly, despite the significant increase of lung metastasis in *PyMT/H2BE^WT/E113K^* mice, there was no change in the overall survival compared to the *PyMT/H2BE^WT/WT^* mice. This could be due to the immense primary tumor burden in the MMTV-PyMT mouse model which could overshadow the impact of lung metastasis and make primary tumor burden the leading cause of mortality in our H2BE113K–MMTV-PyMT breast cancer mouse model. In the clinic, however, metastasis is usually the primary cause of death for breast cancer, while primary tumors are often removable by surgery. Thus, the impact of H2BE113K mutation on lung metastasis may be a significant endpoint for breast cancer research and should not be overlooked.

The H2BE113K knock-in cells exhibited increased enrichment of SMARCA5 at the *G3BP2* promoter region. Moreover, the elevated SMARCA5 enrichment was associated with a more open chromatin structure, which in turn might alter the expression of *G3BP2*. Of note, the enzymatic activity and binding ability of SMARCA5 are essential for its role in nucleosome sliding. Recent studies demonstrated that SMARCA5 mediates nucleosome spacing in vitro (*38*) and regulates chromatin accessibility to facilitate transcription factor binding by sliding nucleosomes near the TSS (*54*–*56*). Therefore, we hypothesis that SMARCA5 may synergistically coordinate with transcription factors in targeting specific genomic loci, regulating chromatin dynamics and modulating gene expression in H2BE113K mutant cells.

To explore potential therapeutic strategies for H2BE113K breast cancer, we applied ED2-AD101, a first-in-class SMARCA5 inhibitor, which effectively diminished the excessive colony formation observed in H2BE113K mutant cells. RNA-seq revealed specific gene up-regulation in untreated H2BE113K mutant cells, which was otherwise suppressed upon ED2-AD101 treatment. Pathway analysis revealed multiple DEGs enriched in cancer-related pathways, including the Wnt signaling pathways. The Wnt pathway is notably dysregulated in multiple cancers and known to promote cell proliferation and stemness. Moreover, the involvement of cellular homeostasis pathways suggests that ED2-AD101 may also impair tumor cell interactions with the microenvironment, a critical factor in cancer progression. Together, these results provide mechanistic insights into the potential anticancer effects of ED2-AD101 in patients with breast cancer harboring H2BE113K mutation.

In summary, our study elucidated the molecular mechanism of H2BE113K in breast cancer. Using CRISPR-Cas9–mediated H2BE113K knock-in breast cancer cells, we demonstrated that the H2BE113K mutation plays a crucial role in enhancing colony formation by altering the expression of cancer-related genes. The FLAG CUT&RUN results revealed that the E113K mutant H2B is enriched at the promoter region of cancer-related genes, where it altered gene expression by increasing chromatin accessibility through regulating SMARCA5 enrichment. Furthermore, we revealed that H2BE113K significantly promotes lung metastasis of breast cancer in vivo by regulating the expression of metastatic-related genes.

## MATERIALS AND METHODS

### Cell culture

Lenti-X cells and MDA-MB-231 cells were cultured with Dulbecco’s modified Eagle’s medium (DMEM; Invitrogen) supplemented with 10% fetal bovine serum (FBS). Cells were cultured at 37°C with 5% CO_2_.

### Mouse model

Male and female C57BL/6 and MMTV-PyMT mice were used in experiments. MMTV-PyMT (strain #022974) mice were originally purchased from the Jackson Laboratory. H2BE113K knock-in mice were offered by Gemparmatech Company. All experimental procedures were approved by the Animal Subjects Ethics Sub-Committee of the City University of Hong Kong (no. A-0581).

### Virus packaging and infection

pVSV-G, pEXQV, and shRNA constructs were transfected with a 1:2:1 ratio using polyethylenimine (PEI; Polysciences, #23966) into Lenti-X. The virus collected at 48 and 72 hours was used to infect MDA-MB-231 cells. Infected cells were selected with puromycin and collected for RNA extraction or ChIP-qPCR.

### CRISPR-Cas9 knock-in cell generation

MDA-MB-231 cells were cultured in DMEM supplemented with 10% FBS. For CRISPR-Cas9–mediated knock-in of the H2BE113K mutation, a repair template (pBlueScript WT/E113K-FLAG donor plasmid) and a lentiCRISPRv2 construct coexpressing the sgRNA and Cas9 were transfected into MDA-MB-231 cells with PEI (DNA:PEI = 1:3). Following 24-hour incubation, cells were subjected to neomycin selection (2 mg/ml). Single clones were isolated and validated through genotyping after 2 weeks of selection to verify integration at the targeted locus. Positive clones were further confirmed by Sanger sequencing.

### Chromatin fractionation

Cells were harvested using trypsin and cold phosphate-buffered saline (PBS), centrifuged at 800 rpm for 5 min at 4°C, and washed twice with cold PBS. The cell pellet was lysed in buffer A [10 mM Hepes (pH 7.9), 10 mM KCl, 1.5 mM MgCl_2_, 0.1% Triton X-100, 10% glycerol, 1 mM dithiothreitol (DTT), and 0.34 M sucrose] for 10 min on ice. The cytoplasmic fraction was obtained by sequential centrifugation at 1300*g* and 20,000*g*. The nuclear pellet was washed three times with buffer A, lysed in buffer B (3 mM EDTA, 0.2 mM EGTA, and 1 mM DTT) for 30 min on ice, and centrifuged at 1700*g* for 5 min at 4°C to separate the soluble chromatin fraction. The insoluble chromatin fraction was washed three times with buffer B, resuspended in PBS, and sonicated at high power for 5 cycles (30 s on, 30 s off). All fractions (cytoplasmic, soluble chromatin, and insoluble chromatin) were boiled at 95°C for 5 min with sample buffer and stored at −20°C for immunoblotting. Protease inhibitors were included in all steps.

### Oncogenic assays

Cells were seeded and cultured for 10 days to evaluate colony formation ability. For cells infected with shRNA virus, puromycin was added into medium to select the infected cells before seeding. After incubation, cells were stained with 0.5% crystal violet to visualize the results. For cell migration, cells were seeded into two chambers with an insert in the middle. Cell positions were photographed before and after insert removal. The migration status was captured using a camera after 10 hours of cell migration. For cell proliferation assay, Cell Counting Kit-8 (CCK8) assay was performed. Cells were seeded and measured under optical density at 450 nm for different time points by a BioTek Synergy H1 Microplate Reader.

### ChIP-qPCR

MDA-MB-231 knock-in cell lines were plated before the assay, fixed with 1% paraformaldehyde (PFA) at room temperature for 5 min, and quenched with 125 mM glycine for 5 min. The fixed cells were washed twice with 1× cold tris-buffered saline and scraped into the extraction buffer [10 mM tris HCl (pH 7.5), 10 mM NaCl, 0.5% NP-40, and protease inhibitor cocktail]. After 30 min of incubation on ice, the lysate was centrifuged at 3000 rpm for 5 min at 4°C. The pellet was washed once with micrococcal nuclease (MNase) digestion buffer [20 mM tris-HCl (pH 7.5), 15 mM NaCl, and 60 mM KCl], resuspended in MNase digestion buffer with 2 mM CaCl_2_, and digested with MNase (New England Biolabs (NEB), #M0247S) at 37°C, 500 rpm for 5 min. The reaction was stopped with 2× stop buffer [100 mM tris-HCl (pH 8.0), 20 mM EDTA, 200 mM NaCl, 2% Triton X-100, and 0.2% sodium deoxycholate], followed by centrifugation at 13,000 rpm for 15 min at 4°C. The supernatant was collected as input samples. The remaining lysate was divided into different fractions and incubated with H2A antibody (Abcam, ab156533)/H3 antibody (Immunoway, YM3038)/FLAG beads (Sigma-Aldrich, F7425) overnight at 4°C, respectively.

Protein G Sepharose 4 Fast Flow (GE Healthcare) beads were added into all fractions and incubated for 3 hours at 4°C. All beads fractions were sequentially washed with ChIP lysis buffer [50 mM Hepes (pH 7.3), 140 mM NaCl, 1 mM EDTA, 1% Triton X-100, 0.1% sodium and deoxycholate], ChIP lysis buffer with 0.5 M NaCl, tris-LiCl buffer [10 mM tris-HCl (pH 8.0), 0.25 M LiCl, 0.5% NP-40, 0.5% sodium deoxycholate, and 1 mM EDTA], and tris-EDTA buffer [50 mM tris-HCl (pH 8.0) and 1 mM EDTA]. DNA was extracted with 10% chelex and eluted with tris-HCl (pH 8.0) buffer. The eluates were clarified at 5000 rpm for 2 min, and the supernatant was subjected to qPCR for quantification.

### RNA-seq for knock-in cells

Ribosomal RNA (rRNA) was removed from total RNA using the NEBNext rRNA Depletion Kit (NEB, E6310), and the rest of the RNA was processed for library preparation using the NEBNext Ultra II Directional RNA Library Prep Kit according to the manufacturer’s instructions. The libraries were validated on the Bioanalyzer 2100 (Agilent) and quantified using the NEBNext Library Quant Kit (NEB, E7630L). The libraries were sequenced on a HiSeq platform (Illumina) using 150-bp paired-end sequencing.

### CUT&RUN-seq

The CUT&RUN assay was adapted from Skene and Henikoff (*57*). Specifically, a total of 5 million cells were collected, washed twice with cold PBS, and pelleted by centrifugation at 600*g* for 3 min at 4°C. Nuclei were isolated by resuspending the cells in NE1 buffer [20 mM Hepes-KOH (pH 7.9), 10 mM KCl, 0.5 mM spermidine, 0.1% Triton X-100, 20% glycerol, and protease inhibitor cocktail] for 10 min. After centrifugation, the nuclei were resuspended in buffer 1 [20 mM Hepes (pH 7.5), 150 mM NaCl, 2 mM EDTA, 0.5 mM spermidine, 0.1% bovine serum albumin, and protease inhibitor cocktail] for 5 min, washed once with buffer 2 [20 mM Hepes (pH 7.5), 150 mM NaCl, 0.5 mM spermidine, 0.1% BSA, and protease inhibitor cocktail], and resuspended in buffer 2. Nuclei were incubated overnight at 4°C with a FLAG antibody (Sigma-Aldrich, F1804) or an H2A antibody (Abcam, ab15653), respectively. For mouse primary antibodies, mouse immunoglobulin G (IgG; AffiniPure rabbit anti-mouse IgG, #315-005-003) was added for 1 hour. The nuclei were then incubated with 150 ng of fusion protein A - MNase (pA-MNase) for 1 hour at 4°C in buffer 2. After washing with buffer 2 and rinse buffer [20 mM Hepes (pH 7.5) and 0.5 mM spermidine], digestion was carried out in digestion buffer [3.5 mM Hepes (pH 7.5), 10 mM CaCl_2_, and protease inhibitor cocktail] on ice for 15 min. The reaction was stopped with 2× stop buffer [40 mM Hepes (pH 7.5), 300 mM NaCl, 40 mM EGTA, 20 mM EDTA, 1 mM spermidine, protease inhibitor cocktail, and 144 pg of yeast spike-in DNA], incubated at 37°C for 30 min, and cleared by centrifugation. DNA was purified using the NucleoSpin Gel and PCR Clean-up Kit and subsequently quantified with a Qubit Fluorometer. Libraries were prepared using the Ovation Ultralow System V2 (NuGEN, #0344NB) and amplified with the PCR program. Libraries were validated using a Bioanalyzer and quantified with the NEBNext Library Quant Kit. Sequencing was performed on a HiSeq X 10, generating approximately 20 million paired-end reads (150 bp).

### ATAC-seq

ATAC-seq was performed as described previously with minor modifications (*58*, *59*). Briefly, 50,000 cells were washed with cold PBS, incubated in cold lysis buffer [10 mM tris-HCl (pH 7.5), 10 mM NaCl, 3 mM MgCl_2_, 0.01% digitonin, 0.1% Tween 20, and 0.1% NP-40] on ice for 3 min, and centrifuged at 500*g* for 3 min. The pellet was washed with washing buffer [10 mM tris-HCl (pH 7.5), 10 mM NaCl, 3 mM MgCl_2_, and 0.1% Tween 20] and centrifuged at 500*g* for 10 min. The cell lysate was resuspended and incubated at 37°C for 30 min in a transposition reaction with 2× TD buffer [20 mM tris-HCl (pH 7.5), 10 mM MgCl_2_, and 20% *N*,*N*-dimethylformamide] and 2.5 μl of Tn5 transposase. DNA was purified using the Qiagen MinElute PCR Purification Kit, and the transposed DNA was amplified by PCR. Libraries were purified, size-selected with AMPure XP beads, assessed using the Bioanalyzer, and quantified with the NEBNext Library Quant Kit. Sequencing was performed on a HiSeq platform with 150-bp paired-end.

### RNA-seq data analysis

Clean reads were aligned to the human genome hg38 and gene annotations from RefSeq gene using STAR v.2.7.7a (*60*). Gene expression was quantified as fragments per kilobase per million using Cufflinks v2.2.1 (*61*). Differential expression analysis between H2BE113K and WT samples was performed using Cuffdiff v2.2.1, with a significance threshold of *P* < 0.01. Annotated genes were grouped into up-regulated, down-regulated, and unchanged genes based on RNA-seq differential expression, and average read coverages for each gene were calculated across the annotated gene regions.

### CUT&RUN-seq data analysis

Yeast chromatin served as a spike-in reference for normalization. FLAG CUT&RUN-seq reads were mapped to a combined human (hg38) and yeast (Sac3) genome according to a previously described procedure (*62*). A custom Bowtie2 library was used with default parameters. Reads were separated by organism, and spike-in reads were used to calculate normalization factors. Peaks were called using MACS2 v2.2.5 (*63*) with a threshold of *P* < 0.001. Read counts within FLAG peaks were counted with BEDTools v.2.29.2 (*64*) and normalized to the normalization factor. Cluster 3.0 software (http://bonsai.hgc.jp/~mdehoon/software/cluster/software.htm), a widely used tool for unsupervised expression pattern analysis, was used with the K-means clustering method—using its default Euclidean distance metric and iterative centroid update algorithm—to group samples and identify potential distinct expression/epigenetic patterns. By using Cluster 3.0, peaks exhibiting a statistically significant increase in Flag CUT&RUN intensity in H2BE113K cells (relative to WT controls) were classified as the G2 subgroup, whereas peaks with conserved signal intensity (showing no significant fold change between H2BE113K and control cells) were designated as the G1 subgroup, respectively. To address the genomic distribution of FLAG CUT&RUN peaks for G1 and G2 groups, peaks in encompassing 3′ untranslated region (3′UTR), transcription termination site, 5′UTR, noncoding, promoter, intron, exon, and intergenic region were annotated using the HOMER tool (http://homer.ucsd.edu/homer/), with default parameter settings applied for consistent peak annotation across all experimental groups.

### ATAC-seq data analysis

ATAC-seq reads were aligned to the human genome (hg38) using Bowtie2 v2.3.4.1 (*65*). Genome-wide coverage was calculated with BEDTools v2.25.0 (*64*) and Perl programs. Peaks were identified using MACS2 v2.2.5 (*63*) with *P* < 0.001. To profile H2BE113K ATAC-seq data, normalized read densities for G1 and G2 peaks were calculated across ±5 kb around peak centers. Up/down/unchanged genes were associated with ATAC-seq signals using the regions upstream and downstream of the TSS (±5 kb), with only the “promoter” regions being covered thereby.

### Tissue isolation and process

The primary tumors and whole lungs were collected individually, photographed, and washed with 1× PBS. Tissues were fixed in 4% PFA at 4°C. A portion of the primary tumors and the entire lungs were paraffin-embedded, sectioned, and processed for H&E and IHC staining by Servicebio Company. For RNA-seq, primary tumors and lung metastatic tumors were immediately flash-frozen in liquid nitrogen to prevent RNA degradation.

### RNA-seq for tumor samples

Tissues were thawed on ice and weighted before RNA extraction. The samples were minced and homogenized using a glass dounce homogenizer. Total RNA was extracted using the MiniBEST Universal RNA Extraction Kit (TaKaRa, #9767). Library preparation and sequencing were performed by Azenta Life Sciences Company.

### In vivo RNA-seq analysis

Raw RNA-seq reads were trimmed, filtered, and assessed using Trim Galore (version: 0.6.10). Then, high-quality reads were mapped to the mouse genome (GRCm38/mm10) using Hisat2 (version: 2.2.1). Read counts were calculated by HTSeq (version: 0.11.3). DESeq2 package was used to perform the different expression analyses for different sample groups (*66*). Reactome gene sets, Kyoto Encyclopedia of Genes and Genomes gene sets, canonical gene sets, and hallmark gene sets were used in the molecular signature analysis (The Molecular Signatures Database (MSigDB) is available at the website of https://www.gsea-msigdb.org/gsea/msigdb/index.jsp).
