# Supplementary material for: H2BE113K mutation promotes breast cancer metastasis through modulating chromatin dynamics
Source: Sci Adv. 2026 Jul 10;12(28):eadx4982. doi: 10.1126/sciadv.adx4982 (PMC13353378; doi:10.1126/sciadv.adx4982)
Supplement: Supplementary file 1 — Figs. S1 to S6 Legends for data S1 to S5 [file sciadv.adx4982_sm.pdf]

Science Advances
AAAS

Supplementary Materials for

H2BE113K mutation promotes breast cancer metastasis through modulating chromatin dynamics

Shiman Hu (胡诗曼) *et al.*

Corresponding author: Haiyun Gan, hy.gan@siat.ac.cn; Kui Ming Chan (陈居明), ming.chan@cityu.edu.hk

Sci. Adv. **12**, eadx4982 (2026)
DOI: 10.1126/sciadv.adx4982

The PDF file includes:

Figs. S1 to S6
Legends for data S1 to S5

Other Supplementary Material for this manuscript includes the following:

Data S1 to S5

Supplementary Figure 1.

a.

Gene	Patient ID	Cancer type	Mutation	Allele frequency
H2BC4	TCGA-BH-A0BV-01	Breast Invasive Ductal Carcinoma	E113K	0.16
H2BC8	TCGA-AO-A03M-01	Breast Invasive Ductal Carcinoma	E113K	0.1
H2BC8	TCGA-OL-A5RW-01	Breast Invasive Ductal Carcinoma	E113K	0.31
H2BC21	MO_1051	Breast Invasive Ductal Carcinoma	E113K	0.13
H2BC21	TCGA-A2-A0EO-01	Breast Invasive Ductal Carcinoma	E113K	0.28
H2BC11	PD4127a	Invasive Breast Carcinoma	E113K	N/A
H2BC3	TCGA-49-AARN-01	Lung Adenocarcinoma	E113K	0.16
H2BC8	nsclc_mskcc_2018s55	Lung Adenocarcinoma	E113K	0.13
H2BC9	TCGA-64-5781-01	Lung Adenocarcinoma	E113K	0.14
H2BC3	TCGA-DK-AA6X-01	Bladder Urothelial Carcinoma	E113K	0.15
H2BC11	WCM259_2	Bladder Urothelial Carcinoma	E113K	N/A
H2BC21	TCGA-YC-A89H-01	Bladder Urothelial Carcinoma	E113K	0.27
H2BC10	TCGA-CV-A45Z-01	Head and Neck Squamous Cell Carcinoma	E113K	0.1
H2BC12	TCGA-BA-5558-01	Head and Neck Squamous Cell Carcinoma	E113K	0.17
H2BC3	TCGA-V5-AASX-01	Esophageal Adenocarcinoma	E113K	0.09
H2BC9	TCGA-AG-4001-01	Rectal Adenocarcinoma	E113K	0.71
H2BC11	TCGA-AZ-6599-01	Colon Adenocarcinoma	E113K	0.2
H2BC14	TCGA-VS-A9UA-01	Cervical Squamous Cell Carcinoma	E113K	0.28
H2BC15	TCGA-OR-A5JB-01	Adrenocortical Carcinoma	E113K	0.35

b.

Gene	Patient ID	Cancer type	Mutation	Allele frequency
H2BC5	P-0044446-T01-IM6	Bladder Urothelial Carcinoma	E113Q	0.25
H2BC8	TCGA-DK-AA75-01	Bladder Urothelial Carcinoma	E113Q	0.38
H2BC8	TCGA-EK-A2PM-01	Cervical Squamous Cell Carcinoma	E113Q	0.36
H2BC9	TCGA-DG-A2KK-01	Cervical Squamous Cell Carcinoma	E113Q	0.37
H2BC11	cscc-ucsf-2021-Sample50	Cutaneous Squamous Cell Carcinoma	E113Q	0.38
H2BC11	YUKLAB	Cutaneous Melanoma	E113Q	N/A
H2BC9	TCGA-CR-7394-01	Head and Neck Squamous Cell Carcinoma	E113Q	0.04
H2BC12	TCGA-CV-A45U-01	Head and Neck Squamous Cell Carcinoma	E113Q	0.19
H2BC4	TCGA-05-5715-01	Lung Adenocarcinoma	E113Q	0.28
H2BC8	TCGA-5P-A9K8-01	Papillary Renal Cell Carcinoma	E113Q	0.25
H2BC9	PD4197a	Invasive Breast Carcinoma	E113Q	N/A
H2BC11	CAC_3700	Ampullary Carcinoma	E113Q	0.43
H2BC17	SP95126	Uterine Endometrioid Carcinoma	E113Q	0.2

c.

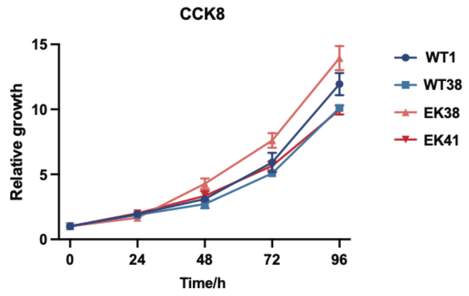

d.

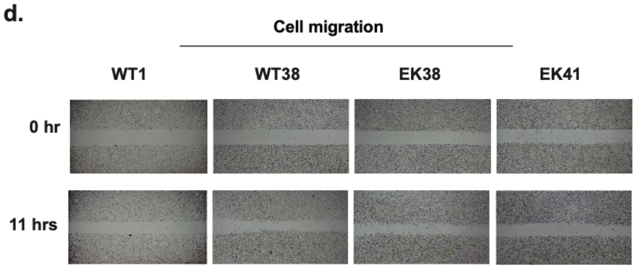

Supp fig. 1 H2BE113K mutant cells did not affect cell proliferation and migration.

a. H2BE113K and b. H2BE113Q cancer patient sample data was obtained from cBioPortal database. c. CCK8 assay revealing the cell proliferation rate of WT and H2BE113K mutant cells. The quantification results of different time points were normalized to 0 hour data. Results from three independent experiments are shown. d. Representative images from three independent experiments showing the cell migration ability of WT and H2BE113K mutant cells.

Supplementary Figure 2.

a.

Upregulated ATAC-seq peaks

Rank	Motif	Name	P value
1		Fos(bZIP)/TSC-Fos-ChIP-Seq(GSE110950)/Homer	1e-1085
2		Fra1(bZIP)/BT549-Fra1-ChIP-Seq(GSE46166)/Homer	1e-1074
3		JunB(bZIP)/DendriticCells-Junb-ChIP-Seq(GSE36099)/Homer	1e-1057
4		Fra2(bZIP)/Striatum-Fra2-ChIP-Seq(GSE43429)/Homer	1e-1053
5		Atf3(bZIP)/GBM-ATF3-ChIP-Seq(GSE33912)/Homer	1e-1031
6		BATF(bZIP)/Th17-BATF-ChIP-Seq(GSE39756)/Homer	1e 1025
7		AP-1(bZIP)/ThioMac-PU.1-ChIP-Seq(GSE21512)/Homer	1e-980
8		Fosl2(bZIP)/3T3L1-Fosl2-ChIP-Seq(GSE56872)/Homer	1e-931
9		Jun-AP1(bZIP)/K562-cJun-ChIP-Seq(GSE31477)/Homer	1e-798
10		CTCF(Zf)/CD4+-CTCF-ChIP-Seq(Barski_et_al.)/Homer	1e 409

Downregulated ATAC-seq peaks

Rank	Motif	Name	P-value
1		Atf3(bZIP)/GBM-ATF3-ChIP-Seq(GSE33912)/Homer	1e-140
2		JunB(bZIP)/DendriticCells-Junb-ChIP-Seq(GSE36099)/Homer	1e-138
3		Fos(bZIP)/TSC-Fos-ChIP-Seq(GSE110950)/Homer	1e 135
4		BATF(bZIP)/Th17-BATF-ChIP-Seq(GSE39756)/Homer	1e-134
5		Fra1(bZIP)/BT549-Fra1-ChIP-Seq(GSE46166)/Homer	1e-133
6		AP-1(bZIP)/ThioMac-PU.1-ChIP-Seq(GSE21512)/Homer	1e-131
7		Fra2(bZIP)/Striatum-Fra2-ChIP-Seq(GSE43429)/Homer	1e-129
8		Fosl2(bZIP)/3T3L1 Fosl2 ChIP Seq(GSE56872)/Homer	1e 118
9		NFkB-p65(RHD)/GM12787-p65-ChIP-Seq(GSE19485)/Homer	1e-104
10		NFkB-p65-Rel(RHD)/ThioMac-LPS-Expression(GSE23622)/Homer	1e-101

b.

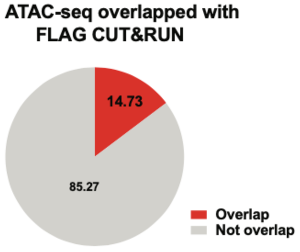

c.

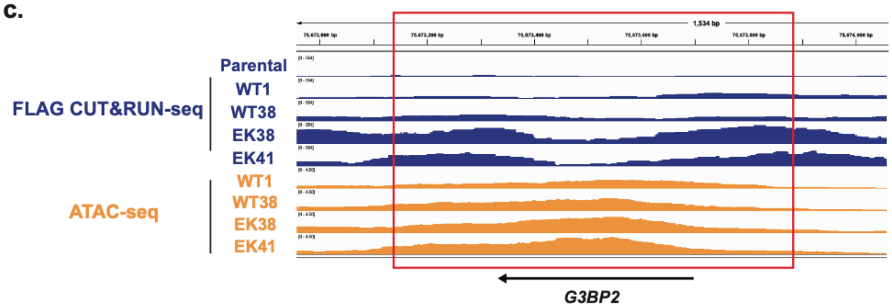

d.

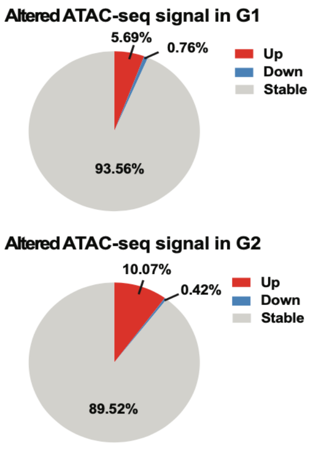

e.

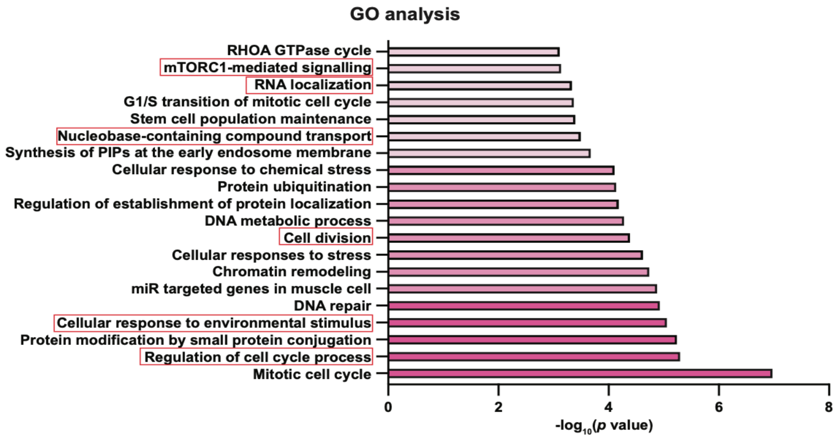

f.

Pathway and functions	Genes
Regulation of cell cycle process	*APBB2, CDKN2C, CENPE, MAD2L1, PKD2, PLRG1, TGFB1, CLOCK, PABIR1 RAD50, CIT, SMC5, ANKRD17, UIMC1, FBXW7, RPTOR, NCAPG, MAP9,*
Cellular response to environmental stimulus	*GNAQ, NINJ1, PKD2, PPID, REST, TGFB1, CLOCK, HABP4, VPS13A, FBXW7, RPTOR, LRRC8C*
Cell division	*CCNG2, CENPC, CENPE, CETN3, STMN1, MAD2L1, MTMR4, PTTG1, CIT, SMC5, NCAPG, MAP9, CNTROB, IQGAP3*
Nucleobase-containing compound transport	*SLC25A4, CETN3, EIF4E, G3BP2, NUP54, METTL14, NUP85, LRRC8C*
RNA localization	*CETN3, EIF4E, G3BP2, NUP54, METTL14, NUP85, NAF1*
mTORC1-mediated signalling	*EIF4E, RPTOR, RRAGC*

Supp fig 2. *G3BP2* was one of H2BE113K targets and was enriched in cancer related pathways.

a. Top 10 enriched transcription factor binding motifs identified in ATAC-seq peaks with increased (left panel) or decreased (right panel) accessibility in H2BE113K mutant cells compared to wild-type. b. Pie chart showing the overlapped percentage between ATAC-seq peaks and FLAG CUT&RUN peaks. c. Genome browser tracks of FLAG CUT&RUN-seq and ATAC-seq data of WT and H2BE113K mutant cells at *G3BP2* gene loci. Regions highlighted in red rectangle represented the position with enriched FLAG signal and increased chromatin accessibility in mutant cells compared to WT cells, respectively. d. Pie chart showing the proportion of up-regulated, down-regulated and stable chromatin accessibility regions in G1 (upper panel) and G2 (lower panel) clusters, respectively. e. Histogram showing top GO terms enriched among 221 candidate genes. GO analysis was performed using a web-based tool (Metascape). f. Table summarizing the cancer-related GO terms from d. and the genes enriched in the corresponding GO terms.

Supplementary Figure 3.

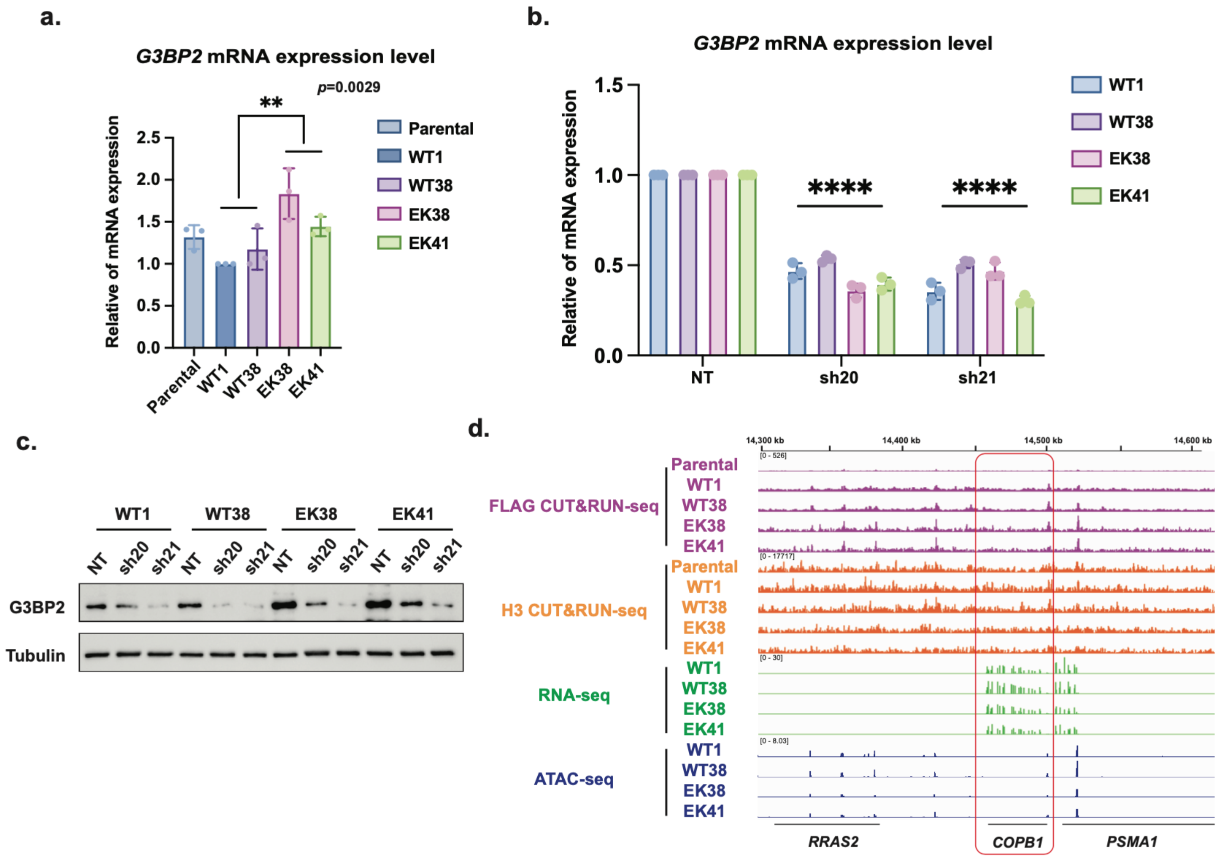

Supp fig 3. *COPB1* had comparable FLAG signal in H2BE113K mutant and WT cells.

a. RT-qPCR analysis showing mRNA levels of *G3BP2* in parental, WT and H2BE113K mutant cells. Results from three independent experiments are shown (mean ± SD, ***p* < 0.01). b. RT-qPCR analysis illustrating mRNA levels of *G3BP2* in WT and H2BE113K mutant cells after shRNA-mediated knockdown. Results from three independent experiments are shown (mean ± SD, *****p* < 0.0001). c. Immunoblotting demonstrating G3BP2 protein levels in WT and H2BE113K knock-in cells after shRNA-mediated knockdown. Representative images from three independent experiments are shown. d. Genome browser tracks of FLAG and H3 CUT&RUN-seq, RNA-seq, and ATAC-seq data at *COPB1* and its nearby gene loci.

Supplementary Figure 4.

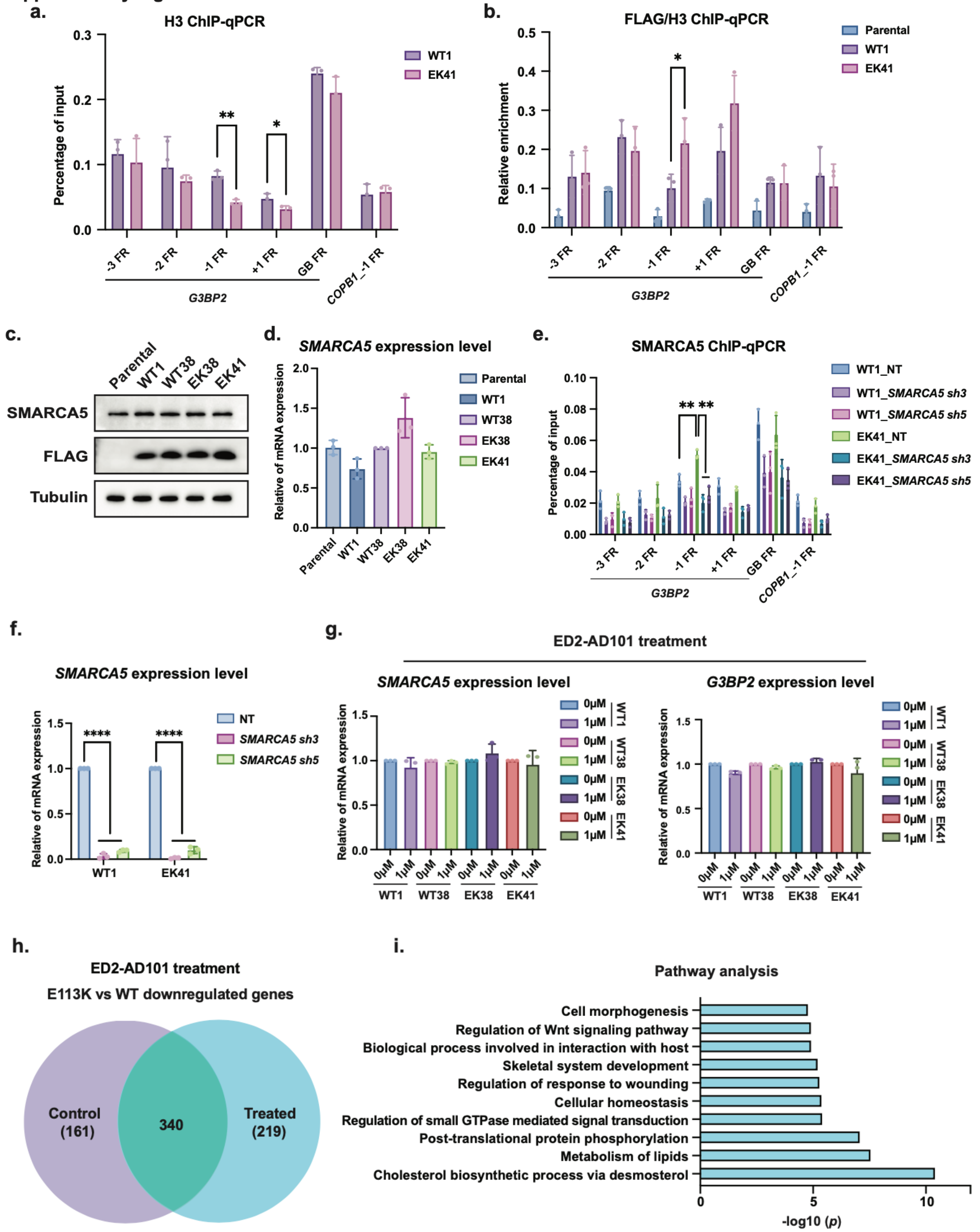

Supp fig 4. ED2-AD101 treatment had no effect on expression level of *SMARCA5* and *G3BP2*. a. ChIP-qPCR analysis of H3 enrichment at various genomic regions, including -3, -2, -1 and +1 nucleosome and GB of *G3BP2* and -1 nucleosome of *COPB1.* Results from three independent experiments are shown (mean ± SD, *p < 0.05, **p < 0.01). b. ChIP-qPCR analysis of relative FLAG enrichment at indicated gene loci. FLAG enrichment over H3 was calculated as fold change relative to H3 ChIP. Results from three independent experiments are shown (mean ± SD, *p < 0.05). c. Immunoblotting revealing SMARCA5 protein expression level in parental and knock-in cells. Representative images from three independent experiments are shown. d. RT-qPCR analysis showing mRNA expression level of *SMARCA5* in WT and H2BE113K mutant cells. Results are shown as mean ± SD from three independent experiments. e. ChIP-qPCR analysis of SMARCA5 enrichment at various genomic regions, including -3, -2, -1 and +1 nucleosome and GB of *G3BP2* and -1 nucleosome of *COPB1.* Results from three independent experiments are shown (mean ± SD, **p < 0.01). f. RT-qPCR analysis revealing mRNA levels of *SMARCA5* in WT and H2BE113K mutant cells after shRNA-mediated knockdown. Results from three independent experiments are shown (mean ± SD, ****p < 0.0001). g. RT-qPCR analysis demonstrating mRNA levels of *SMARCA5* and *G3BP2* after ED2-AD101 treatment. Results are shown as mean ± SD from three independent experiments. h. Venn diagram demonstrating the numbers of downregulated genes in H2BE113K mutant cells in the control or ED2-AD101 treated group. i. Histograms revealing the top gene ontology (GO) terms and KEGG pathways enriched among the 219 genes specifically downregulated in the H2BE113K mutant cells compared to WT cells from ED2-AD101 treated group. *p*-values were calculated by hypergeometric test.

Supplementary Figure 5.

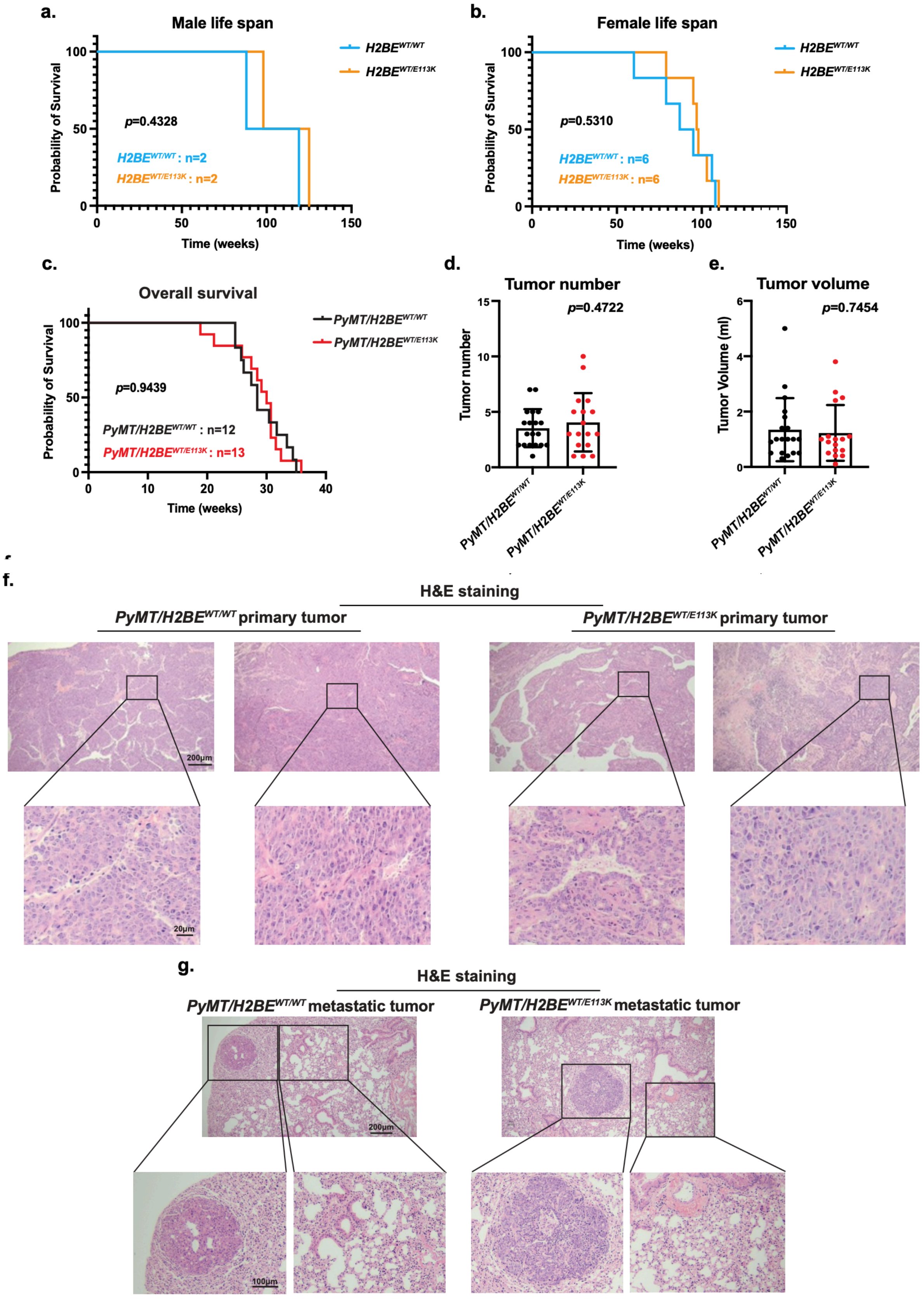

Supp fig 5. H2BE113K did not alter the morphology of mammary tumors.

a. Kaplan–Meier (KM) survival curves of male and b. female *H2BE*$^{WT/WT}$ and *H2BE*$^{WT/E113K}$ mice. For each group of male mice, n=2; for each group of female mice, n=6. *p* values were calculated using log-rank test. c. Kaplan–Meier (KM) survival curves of female *PyMT/H2BE*$^{WT/WT}$ (n=12) and *PyMT/H2BE*$^{WT/E113K}$ mice (n=13). *p* value was calculated using log-rank test. d. Tumor number and e. tumor volume were measured and analyzed in the *PyMT/H2BE*$^{WT/WT}$ (n=19) and *PyMT/H2BE*$^{WT/E113K}$ (n=17) mice. *p* values were calculated by unpaired, two-tailed Student *t*-test. f. Representative H&E staining of primary tumors harvested from *PyMT/H2BE*$^{WT/WT}$ and *PyMT/H2BE*$^{WT/E113K}$ mice. Scale bar, 200 µm (upper, zoom out); 20 µm (lower, zoom in). e. Representative H&E staining images of lung metastatic tumors harvested from *PyMT/H2BE*$^{WT/WT}$ and *PyMT/H2BE*$^{WT/E113K}$ mice. Scale bar, 200 µm (upper, zoom out); 100 µm (lower, zoom in).

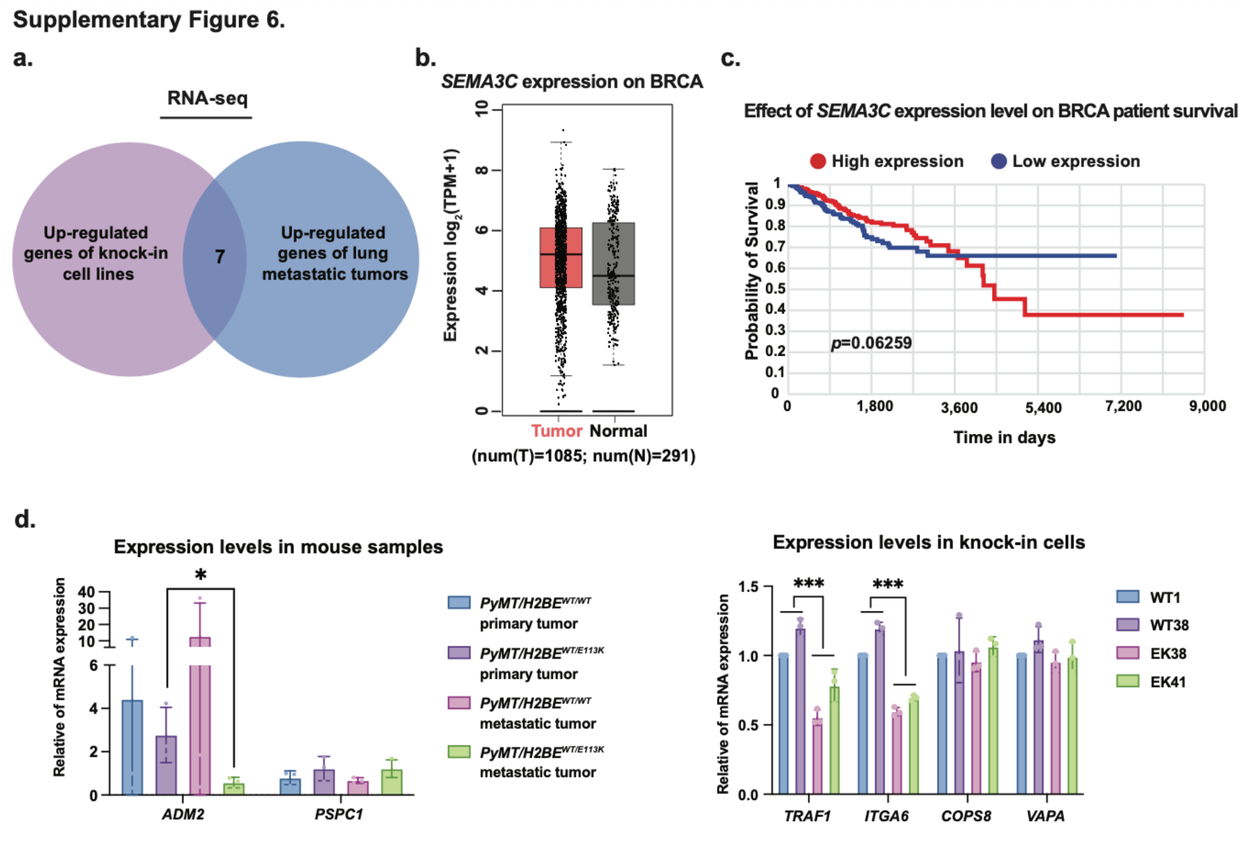

Supp Fig 6. Elevated expression of *SEMA3C* was correlated with poor prognosis in breast cancer patients.

a. Venn diagram demonstrating the numbers of common upregulated genes between the RNA-seq of lung metastatic tumor samples and knock-in cell lines. b. Expression level of *SEMA3C* in tumors and normal tissues of BRCA patients. Data was extracted from TCGA database and plotted through GEPIA online tool. c. Kaplan–Meier (KM) plot showing *SEMA3C* expression-based overall survival analysis. The KM plot was generated by a web-based tool OncoLnc on breast cancer patients. d. RT-qPCR analysis revealing mRNA levels of indicated genes. *ADM2* and *PSPC1* served as controls in tumor samples. *TRAF1*, *ITGA6*, *COPS8*, and *VAPA* served as controls in knock-in cells. Results from three independent experiments are shown (mean ± SD, $*p < 0.05$, $***p < 0.001$). p value was calculated by unpaired, two-tailed Student t-test.

Titles of supplementary excel files
Data S1, counts table for RNA-seq (inhibitor treatment)
Data S2, counts table for RNA-seq (knock-in cell lines)
Data S3, counts table for RNA-seq (tumor samples)
Data S4, bed files (ATAC-seq, CUT&RUN, intersection of ATAC-seq and CUT&RUN)
Data S5, overlapped candidate gene list
